# Demeton methyl: MAK Value Documentation, addendum – Translation of the German version from 2024

**DOI:** 10.34865/mb802200e11_2ad

**Published:** 2026-06-30

**Authors:** Andrea Hartwig, MAK Commission

**Affiliations:** 1 Institute of Applied Biosciences, Department of Food Chemistry and Toxicology, Karlsruhe Institute of Technology (KIT) Adenauerring 20a, Building 50.41 76131 Karlsruhe Germany; 2 Permanent Senate Commission for the Investigation of Health Hazards of Chemical Compounds in the Work Area, Deutsche Forschungsgemeinschaft, Kennedyallee 40, 53175 Bonn, Germany. Further information: Permanent Senate Commission for the Investigation of Health Hazards of Chemical Compounds in the Work Area | DFG

**Keywords:** demeton methyl, insecticide, pesticide, toxicity, evaluation, acetylcholinesterase inhibitor

## Abstract

Demeton methyl [8022-00-2] is used as an insecticide but is no longer approved in the European Union. The previous evaluation does not reflect the current data situation of the substance. The MAK Commissiondecided that a new evaluation is not of high priority. The MAK value and the other classifications are therefore suspended and the substance is listed in the Section II c of the List of MAK and BAT Values for substances no longer evaluated.

**Table:** 

**MAK value**	**see Section II c of the List of MAK and BAT Values**
**Peak limitation**	**–**
	
**Absorption through the skin**	**–**
**Sensitization**	**–**
**Carcinogenicity**	**–**
**Prenatal toxicity**	**–**
**Germ cell mutagenicity**	**–**
	
**BLW (2023)**	**reduction of the acetylcholinesterase activity in erythrocytes to 70% of the reference value^a)^**
	
Synonyms	methyl mercaptophos *S*-[2-(ethylsulfanyl)ethyl]-*O*,*O*-dimethylphosphorothioate *O*-[2-(ethylsulfanyl)ethyl]-*O*,*O*-dimethylphosphorothioate metasystox
Chemical name (IUPAC)	1-dimethoxyphosphorylsulfanyl-2-ethylsulfanylethane 2-ethylsulfanylethoxydimethoxy-sulfanylidene-λ^5^-phosphane
CAS number	8022-00-2
Molar mass	230.28 g/mol
Melting point	< 25 °C (RSC 2022)
Boiling point	decomposes (ILO and WHO 2021)
Density at 20 °C	1.2 g/cm^3^ (ILO and WHO 2021)
Vapour pressure at 20 °C	0.0004 hPa (ILO and WHO 2021)
log K_OW_	1.32 (ILO and WHO 2021)
Solubility	22 g/l water (NCBI 2022)
**1 ml/m^3^ (ppm) ≙ 9.555 mg/m^3^**	**1 mg/m^3^ ≙ 0.105 ml/m^3^ (ppm)**

^a)^ The BLW (biological guidance value) is derived as the ceiling value because of acute toxic effects.

This addendum was prepared because the previous evaluation no longer reflects the data currently available for the MAK value and for the designations and classifications of the substance. Demeton methyl is a mixture of isomers made up of demeton-*O*-methyl [867-27-6] and demeton-*S*-methyl [919-86-8]. Demeton methyl is an insecticide from the class of organophosphates. It inhibits acetylcholinesterase. The biological guidance value (BLW) for acetylcholinesterase inhibitors (reduction of the acetylcholinesterase activity in erythrocytes to 70% of the reference value; Lewalter 1995; Weistenhöfer et al. 2024) therefore applies to demeton methyl. The BLW is derived as the ceiling value because of acute toxic effects. However, it was not investigated whether this is the most sensitive end point.

A MAK value of 0.5 ml/m^3^ was established in 1958 based on the TLV value set by ACGIH at the time. Demeton methyl was designated with an “H” (for substances which can be absorbed through the skin in toxicologically relevant amounts). In 2002, the substance was assigned to Peak Limitation Category II with an excursion factor of 2 (Greim 2002, available in German only). To date, there is no MAK value documentation for demeton methyl.

The isomers demeton-*O*-methyl and demeton-*S*-methyl are used to control sucking insects (AERU 2022 a, b). The *S* isomer was approved for use in the Federal Republic of Germany between 1971 and 1994; there was no approval for the *O* isomer. In the former German Democratic Republic, both isomers were approved until 1967 (BVL 2010). No plant protection products containing these isomers as active substances are approved in the EU; the entry for the *S* isomer has lapsed and no authorization was ever granted for the *O* isomer (European Parliament and European Council 2009; Lewis et al. 2016 a, b).

The previous evaluation does not reflect the currently available data. However, a re-evaluation of the substance is not a priority. Therefore, the MAK value, the peak limitation and the “H” designation have been withdrawn and demeton methyl has been allocated to Section II c of the List of MAK and BAT Values (DFG 2022). This section lists substances for which the previous MAK values, designations and classifications have been withdrawn and which are no longer being reviewed at present.
